# Editorial: Novel Therapeutic Approaches in Inflammatory Bowel Diseases

**DOI:** 10.3390/biomedicines11092466

**Published:** 2023-09-05

**Authors:** Giovanni Pallio

**Affiliations:** Department of Biomedical and Dental Sciences and Morphological and Functional Imaging, University of Messina, Via C. Valeria, 98125 Messina, Italy; gpallio@unime.it

Inflammatory bowel diseases (IBDs) encompass ulcerative colitis (UC) and Crohn’s disease (CD), both of which are inflammatory ailments affecting the gastrointestinal tract. Despite their prevalence, the exact causes of IBDs are yet to be fully understood. However, mounting evidence points to the possibility that these conditions might emerge due to changes in immunological, environmental, psychological, and genetic elements. UC presents as recurring inflammation that is limited to the colon, lacking transmural involvement. Its hallmark indications consist of bloody diarrhea and abdominal cramps primarily occurring during defecation. In contrast, CD represents recurring transmural inflammation of the gastrointestinal lining, affecting the entire digestive tract in a segmented pattern from the oral cavity to the anus. The primary clinical manifestations of CD encompass diarrhea, abdominal discomfort, and fever [1]. IBDs are linked to an imbalance between reactive oxygen species (ROS) and the antioxidant response, resulting in oxidative stress, which is deemed a pivotal element in the origin, advancement, and seriousness of IBDs. The escalation in ROS prompts the activation of nuclear factor-κB (NF-κB), leading to an excessive generation of interleukin-1β (IL-1β) and Tumor Necrosis Factor-alpha (TNF-α). Furthermore, the oxidation of membranes releases arachidonic acid, which is then transformed into eicosanoids, intensifying the harm and amplifying the expression of adhesion molecules like Intercellular Adhesion Molecule 1 (ICAM-1) and cell infiltration. This cascade culminates in the apoptosis of epithelial cells and damage to the mucosa. Standard treatment for IBDs relies on anti-inflammatory medications, corticosteroids, immunosuppressants (e.g., methotrexate), and biologic drugs. However, the limited rate of remission and the significant adverse effects associated with prolonged use of these treatments are unsatisfactory [2]. For this reason, there is great interest in finding new therapeutic strategies with a better safety profile targeting the multifactorial recurrent inflammatory cascade present during chronic intestinal inflammation. The research articles and reviews collected in the three editions of this Special Issue present significant insights into the pathogenesis, molecular pathways, and beneficial effects of novel and safe treatments for IBDs.

Shastri and colleagues investigated the impacts of Idebenone, a short-chain quinone drug possessing anti-inflammatory, antioxidant, and mitochondrial electron-donating properties, in Winnie mice. These mice are known for their spontaneous, chronic intestinal inflammation and endoplasmic reticulum (ER) stress due to a missense mutation in the MUC2 gene encoding mucin. Idebenone administration led to notable enhancements in parameters including body weight gain, disease activity index (DAI), colon length, and histopathology score. Additionally, there was a rise in MUC2 mRNA and protein levels, accompanied by a decrease in expression of ER stress indicators such as C/EBP homologous protein (CHOP), activating transcription factor 6 (ATF6), and X-box binding protein-1 (XBP-1), both at the mRNA and protein levels. The treatment also effectively diminished the levels of pro-inflammatory cytokines in the colon, indicating that Idebenone could offer a promising therapeutic avenue for UC due to its potent anti-inflammatory activity and its capability to alleviate markers of ER stress [1]. The potential targeting of oxidative stress and the inflammatory process in IBDs was also explored in two additional papers within this Special Issue. Pallio et al. and Kand et al. both undertook investigations in this direction. In the first study, Pallio and colleagues delved into the impact of a combination of polynucleotides and hyaluronic acid using an experimental model of dinitrobenzenesulfonic acid (DNBS)-induced colitis. This treatment led to a reduction in clinical symptoms, weight loss, and colon shortening. It also ameliorated macroscopic and histological damage, as well as apoptosis, thereby decreasing the presence of CD3-positive T cells and CD20-positive B cells within the colonic infiltrate. Moreover, this combination led to a decrease in colonic myeloperoxidase activity and malondialdehyde, indicating a mitigation of both inflammatory infiltrates and oxidation-related products [2]. In the second study, Kang and colleagues showed the anti-inflammatory effects of extracellular vesicles derived from Lactobacillus kefirgranum PRCC-1301 (PRCC-1301 EVs) in both acute and chronic murine colitis models. These EVs derived from PRCC-1301 mitigated the loss of body weight, colon shortening, and histological damage. Additionally, they reduced the expression of phosphorylated NF-κB p65 within colon tissue in both the acute and chronic colitis models in mice [3]. These results collectively suggest that the combined treatment of polynucleotides with hyaluronic acid as well as the PRCC-1301 EVs had a protective effect during UC and could be an alternate treatment option for IBD patients. Joo and colleagues took a different approach, investigating immune regulation’s role in IBDs. Specifically, they unveiled that extracellular vesicles from Thapsigargin-Treated Mesenchymal Stem Cells (TSG-EV) could alleviate experimental colitis. This was achieved through a reduction in the inflammatory response and maintenance of intestinal barrier integrity, facilitated by enhanced immunomodulatory properties. Evidence came in the form of increased Tregs and M2-type macrophages in the colons of TSG-EV-treated mice. These findings suggest that TSG-EV holds promise as a novel therapeutic avenue for colitis due to its potent immunomodulatory effects [4].

In the second edition of this Special Issue, most of the papers aimed to investigate the potential anti-inflammatory effects of several compounds. Within this context, ElMahdy and colleagues studied the impact of Dapagliflozin (DAPA) in a mouse model of UC. DAPA exhibited significant reductions in the colon/body weight index, colon weight/colon length ratio, and macroscopic UC scoring. It also maintained the histopathological tissue architecture; suppressed inflammatory biomarkers including colon MCP1, IL-18, Caspase-3, and NF-κB; and successfully restored oxidant/antioxidant balance. These findings collectively suggest that DAPA could be a promising approach for ameliorating UC through its antioxidative, anti-inflammatory, and antiapoptotic properties [5]. Silva et al. aimed to assess the effectiveness and safety of Hemin (a heme-oxygenase inducer) in a chronic 2,4,6-trinitrobenzenesulfonic acid (TNBS)-induced colitis model in rodents. Their study demonstrated hemin’s ability to decrease TNF-α levels, fecal calprotectin, and fecal hemoglobin. Importantly, Hemin was deemed safe in terms of extraintestinal manifestations, showing no adverse effects on the kidneys or liver. As a result, the authors concluded that Hemin treatment could represent a novel pharmacological approach to managing IBD [6]. Kim and colleagues evaluated the effectiveness of inhibiting Sirtuin 7 (SIRT7) in a colitis mouse model. The study revealed that inflammatory cytokines and mucosal inflammation extent were suppressed in colitis-induced mice treated with the SIRT7 inhibitor, indicating SIRT7 as a potential novel target for IBD treatment [7]. Furthermore, high-quality clinical trials were published in the second edition of this Special Issue. Mocci et al. demonstrated that ADA biosimilar GP2017 (HyrimozTM) and its originator (HumiraTM) were equivalent in terms of their efficacy and safety for IBD patients [8]. Huguet et al. indicated that the subcutaneous formulation of Infliximab (CT-P13) achieved higher drug levels, less immunogenicity, better perianal disease control, and improved mucosal healing compared to intravenous Infliximab [9]. Dipasquale and colleagues demonstrated that the enhanced recovery after surgery (ERAS) protocol significantly reduced postoperative complication rates and the timing of first defecation compared to traditional perioperative regimens, suggesting its superiority [10]. Finally, Papa et al. investigated the impact of SARS-CoV-2 infection on the course of IBDs in patients treated with biological agents, revealing that SARS-CoV-2 infection did not increase IBD recurrence rates in this patient cohort [11]. Moreover, the second edition of this Special Issue features high-quality reviews, systematic reviews, and meta-analyses that provide significant updates on novel and safe treatments for gastrointestinal disorders. Vinci et al. conducted a meta-analysis demonstrating that cannabinoid supplementation as an adjuvant therapy might enhance the success of standard therapy for CD, while its usage in UC is not recommended [12]. Likewise, Ashizuka and colleagues comprehensively described the ability of the bioactive peptide Adrenomedullin to suppress inflammatory cytokines production, promote vascular and lymphatic regeneration, enhance mucosal epithelial repair, and boost immune function in various animal models of IBD [13]. Similarly, Yusuf et al. thoroughly elucidated the significance of appropriate fiber intake in managing IBDs, highlighting its substantial benefits for IBD patients [14]. Moreover, Del Buono and colleagues outlined the utilization of oral modulators targeting the sphingosine 1-phosphate (S1P) receptors (including ozanimod, etrasimod, fingolimod, and laquinimod) in patients with IBDs. The majority of S1P modulators have exhibited safety and efficacy in addressing both UC and CD. These compounds demonstrate notable rates of achieving clinical remission, endoscopic improvement, and remission, with minimal safety concerns. However, it should be noted that the interaction of S1P with two of its G-protein-coupled receptors, namely S1PR2 and S1PR3, could potentially be linked to cardiovascular risks [15]. Furthermore, Kelm and colleagues provided evidence that surgical interventions, such as ileocecal resection (ICR), could serve as a promising therapeutic avenue for addressing localized terminal ileitis. Early resection has been shown to enhance quality of life and substantially diminish the requirement for postoperative immunosuppressive medications, maintaining low rates of morbidity. Moreover, novel surgical strategies like Kono-S anastomosis and the incorporation of the mesentery have led to notable reductions in disease recurrence and the need for reoperation. These findings underscore the significance of surgery as a viable alternative to medical treatment in the comprehensive management of patients with CD [16]. Finally, Lauro et al. and Hahn et al. highlighted the role of genetic factors in IBD treatment, indicating that pre-treatment genotyping/sequencing should be incorporated into clinical IBD management guidelines, as this is the most appropriate strategy of selecting the most suitable biological drug for an individual patient [17,18].

In the third edition of this Special Issue, both preclinical and clinical studies were published. Kangwan et al. investigated the effects of Proanthocyanidin-rich red rice extract (PRRE) in dextran sulfate sodium (DSS)-induced colitis mice. PRRE exhibited a notable improvement in the severity of colitis induced by DSS, evident from the reduction in the daily activity index and the restoration of colon length. Administering PRRE led to a significant decrease in the histopathological index and the presence of inflammatory cells. Additionally, the application of PRRE effectively enhanced the presence of mucus in colonic goblet cells, as revealed by PAS staining. Moreover, PRRE treatment successfully suppressed the production of pro-inflammatory cytokines (TNF-α, IL-1β, and IL-6) induced by DSS. In conclusion, these findings indicate that PRRE could be developed as a natural active pharmaceutical component for IBDs [19]. Another two papers focused on real-life IBD patient data demonstrated that early clinical remission can be used to predict long-term remission when using Vedolizumab for UC patients [20] and that in the initial year of treatment, Ustekinumab and Adalimumab exhibited a greater rate of persistence. Subsequently, Adalimumab maintained the highest persistence rate throughout the fourth year. However, when considering the fifth year, those who were most inclined to continue treatment were individuals utilizing Infliximab [21]. Furthermore, in the third edition of this Special Issue, high-quality reviews, systematic reviews, and meta-analysis present relevant updates of data on novel and safe treatments in gastrointestinal disorders. In this context, Atagozli et al. showed that helminths or helminth products could be attractive as novel therapeutic approaches to treat IBDs in light of their ability to stimulate T helper-2 (Th2) and their safety profile, suggesting that clinical research on helminths could lead to the development of safe, potent, and novel therapeutic approaches to preventing or treating IBDs [22]. A systematic review conducted by State et al. highlighted that the criteria for a response from both patients with UC and CD are heterogenous; endoscopic response and outcomes are assessed at variable time points; and current society guidelines provide heterogenous recommendations on treatment optimization. This lack of clear definitions and formal recommendations could lead to empirical treatment strategies and premature abandonment of therapies [23]. Finally, Zurba and colleagues conducted a very elegant review on the state of the art in IBD treatment. They highlighted novel therapeutic target agents in phases II and III of development, such as S1P receptor modulators, selective Janus kinase inhibitors, anti-interleukins, and other small molecules currently under research, as well as emerging treatments for CD and UC that have just received approval or are undergoing phase III clinical trials. The authors observed that several novel therapeutic targets have been identified and studied by developing small molecules and biologic agents that work on one or several combined targets. However, selecting the most suitable medication for each patient has become a challenge due to the increasing range of therapeutic options for treating IBD [24].

In conclusion, all these observations underscore the need to adopt a precision medicine approach which considers patient demographics, medical history, response predictors, preferred administration methods, and disease characteristics in order to determine an effective management strategy.
